# The accuracy of clinical malaria case reporting at primary health care facilities in Honiara, Solomon Islands

**DOI:** 10.1186/1475-2875-8-80

**Published:** 2009-04-23

**Authors:** Ayano Kunimitsu

**Affiliations:** 1 School of Public Health, University of California, Los Angeles, CA, USA; 2 Department of Health Policy Science, Tokyo Medical and Dental University, Tokyo, Japan

## Abstract

**Background:**

The accuracy of malaria case reporting is challenging due to restricted human and material resources in many countries. The reporting often depends on the clinical diagnosis because of the scarcity of microscopic examinations. Particularly, clinical malaria case reporting by primary health care facilities (local clinics), which constitutes the baseline data of surveillance, has never previously been sufficiently evaluated. In order to improve the malaria reporting system to the level required to eventually eliminate this disease, this study estimates the gaps between the records of clinics and government statistics regarding the incidence of clinical malaria, and then also examines some factors that might explain the data discrepancy, including such variables as clinic staffing and record keeping.

**Methods:**

All medical records for outpatients in 2007, handwritten by nurses, were collected from local clinics in Honiara, the capital of the Solomon Islands. The all-monthly clinical malaria cases were then recalculated. The corresponding monthly data in official statistics were provided by the government. Next, in order to estimate any data discrepancy, the ratio of the cases recorded at clinics to the cases reported to the government was determined on the monthly basis. Finally, the associations between the monthly discrepancy and other variables were evaluated by a multiple regression analysis.

**Results:**

The mean data discrepancy between the records of clinics and government statistics was 21.2% (n = 96). Significant associations were observed between the discrepancy and the average number of patients (coefficient: 0.05, 95%CI: 0.31, 0.07), illegible handwriting (coefficient: 0.09, 95%CI: 0.04, 0.15), the use of tally sheets (coefficient:-0.38, 95%CI: -0.54, -0.22), and the clinic level (coefficient:-0.48, 95%CI:-0.89,-0.06).

**Conclusion:**

The findings of this study demonstrate the huge data discrepancy between the records of clinics and government statistics in regard to clinical malaria case reporting. Moreover, the high numbers of patients, illegible writing, the disuse of tally sheets, and insufficient resources at some clinics are likely to be related to the increase in the discrepancy. The clinical malaria case reporting at the local clinic level therefore urgently needs improvement, in order to achieve both better malaria surveillance and to also eventually eliminate this disease in the Solomon Islands.

## Background

Malaria case reporting at primary health care facilities, which constitute the baseline data of the surveillance, is currently being carried out in most countries and the collected epidemiological information is submitted to the World Health Organization (WHO) [1]. Malaria reporting from national surveillance systems, however, varies in quality and reporting completeness [1,2]. The inadequate surveillance may over or underestimate the actual malaria burden. In addition, many developing countries have a scarcity of microscopists, which makes the detection of malaria cases often dependent on clinical suspicion by nurses [1,3]. Such clinical diagnosis, however, is less accurate than microscopic diagnosis, a gold standard for malaria diagnosis, which even has the need for uniformed training, quality control, and standardized reporting methods [1,3-5]. In order to improve malaria surveillance and achieve malaria elimination, the accuracy in both of diagnosis and reporting may be compulsory issues. The diagnostic accuracy of clinical malaria in primary health care facilities was evaluated by Font *et al *as the sensitivity of 70.4% (95% confidence interval; 65.9–74.8%) and the specificity of 68.9% (95% confidence interval; 66.2–71.5%) in Tanzania [3]. On the other hand, the reporting accuracy to investigate whether original cases in clinics are correctly reported to countries is still in doubt.

**Figure 1:**
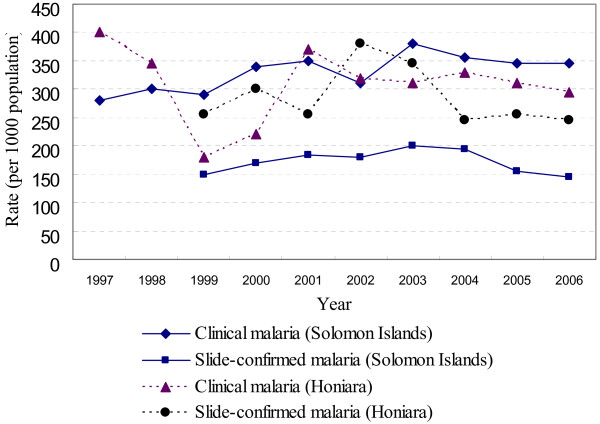
**Malaria incidence in Solomon Islands and Honiara, the capital (1997–2006)**.

In the Solomon Islands (SI), in the south-western Pacific, malaria is still one of the leading causes for morbidity with high incidence rate (Figure 1), hence malaria elimination is a part of main goals in public health for SI [Annual Health Report 2006 Solomon Islands. National Health Statistics Office, Division of Policy and Planning, Ministry of Health, Solomon Islands. 2007 (unpublished); Solomon Islands Health Institutional Strengthening Project (HISP). Solomon Islands National Health Review. 2006 (unpublished)]. Several measures for malaria elimination in SI have already been taken by the government cooperated with supporting donors and recently established the Malaria Elimination Group (MEG) comprised of experts from all over the world [Annual Health Report 2006 Solomon Islands. National Health Statistics Office, Division of Policy and Planning, Ministry of Health, Solomon Islands. 2007 (unpublished); Solomon Islands Health Institutional Strengthening Project (HISP). Solomon Islands National Health Review. 2006 (unpublished)] [6-8]. SI, however, has struggled to accurately report malaria cases because of restrictions in both human and material resources for the reporting system.

**Figure 2:**
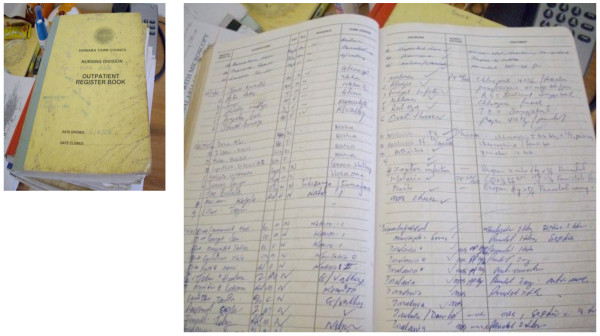
**"Outpatient books" (outpatient records) handwritten by nurses in local clinics. Honiara, Solomon Islands, 2008**.

**Figure 3:**
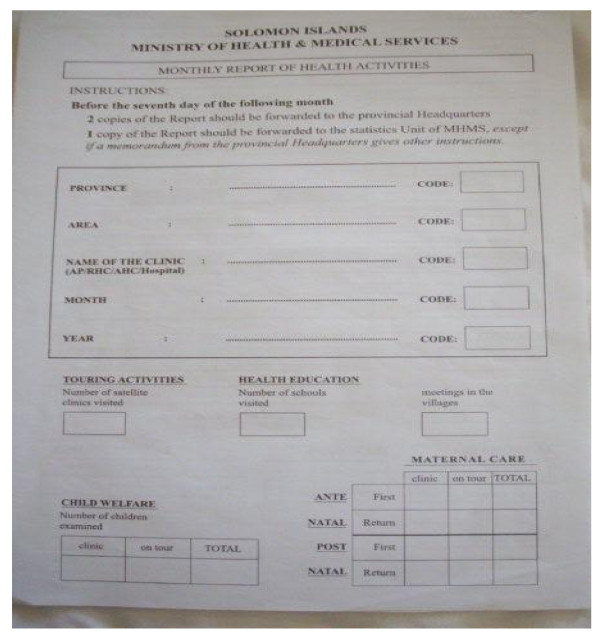
**"Monthly report" for case reporting submitted by local clinics to the central government. Honiara, Solomon Islands, 2008**.

In SI, clinical malaria case reporting has been organized by Health Information System (HIS) in the Ministry of Health of the central government, which collects monthly incident clinical malaria cases from all primary health care facilities (clinics) through each province. In the HIS, clinical malaria is defined by the government as "all patients with symptoms of malaria (confirmed and presumptive) who are treated by anti- malarial drugs". Symptoms of malaria taken into consideration are fever > 37.5°C, a history of fever, and clinical evidence of malaria [Annual Health Report 2006 Solomon Islands. National Health Statistics Office, Division of Policy and Planning, Ministry of Health, Solomon Islands. 2007 (unpublished)]. At the clinic level, nurses handwrite patients' information into "outpatient books" (Figure 2), which summarizes all patients' names, sex, age, addresses, diagnosis, investigation (slide-confirmation for malaria) and treatment. The reporting process of clinical malaria based on the outpatient books in clinics is the following. At the end of every month, according to the records in outpatient books, nurses fill in a "monthly report" to report cases including clinical malaria to the central government (Figure 3). The reporting form is determined by the government. Then, paper copies of the monthly reports are sent from each clinic to the Ministry of Health through each province. In the submission, it is supposed that officers in both province and government give feedback to clinics if the numbers of cases are suspected to be strange, and then confirm the endemic status. But it has been pointed that the feedback does not always work well, since there is no official bulletin about feedback, which totally depends on the officers' impression. Finally, officers at statistics division in the Ministry of Health input the number of cases into HIS system for data compilation of national figures for SI, which is finally reported to WHO.

Therefore, the clinics' data for clinical malaria cases can be definitely key records for malaria surveillance in SI. The reporting accuracy to verify the errors such as misreporting and miscounting occurred in the clinics, however, has never previously been researched. Moreover, as for the reporting, supervision and training to nurses has been insufficient. Thus, in order to improve the malaria reporting system, which provides the baseline data of the surveillance, to a level required for the elimination in SI, this study evaluated the accuracy of clinical malaria data reporting at clinic level. For the evaluation of the accuracy, gaps between clinics' records and government statistics were estimated. And then some factors that might explain this discrepancy, including variables such as clinic staffing and record keeping, were examined.

## Methods

### Study area and population

The study was conducted in July and August 2008 in Honiara, the capital city of SI. Honiara was chosen because the overall infrastructure allows research to be carried out compared to other provinces that suffer greatly from scarcity of reporting resources. According to the government statistics showing percentages of monthly reports that the government received from local clinics through each province, Honiara had 100% completeness of Health Information System (HIS) reporting in 2007 (i.e., every local clinic in Honiara submitted the report every month), although total in SI was 60% [1] [Annual Health Report 2006 Solomon Islands. National Health Statistics Office, Division of Policy and Planning, Ministry of Health, Solomon Islands. 2007 (unpublished)]. Also, clinical malaria incidence rate in Honiara had been smaller than in the total of SI (300 vs. 350 per 1,000 population in 2006), though confirmed malaria in Honiara had larger rate than in SI (250 vs. 150 per 1,000 population in 2006) (Figure 1), which implies that Honiara has more microscopists to make confirmation. Another merit is that, in Honiara, most local people with high risk of malaria use only local health clinics, while some provinces have private charity clinics used by local people and not included in HIS.

There are eight clinics in Honiara: Kukum, Mataniko Mbokona, Mbokonavera, Naha, Rove, Vura, and White river. Each clinic has some registered nurses with specialized education, nurse aides helping registered nurses, and microscopists who give confirmations for malaria diagnosis. Usually they rotate into each clinic at the beginning of the year but sometimes in the middle of the year. Two clinics, Kukum and Rove, are upper clinics called "Area Health Clinics (AHC)"given more human and material resources by the government compared to other six clinics called "Urban Health Clinics (UHC)". AHC are expected to work as "gate keepers" before patients are referred to the central hospital in Honiara. The population covered by clinics in Honiara is likely to be much larger than the actual population of Honiara, about 59,060 estimated people in 2007(unpublished data in the ministry of finance and treasury, SI), since people in other provinces also often visit clinics in Honiara due to the insufficient conditions of clinics in rural area. In Honiara, the HIS system including clinical malaria case reporting has been supervised by Honiara city council as province and the central government.

### Case definition and data collection

In eight clinics at Honiara, all of outpatient books in 2007 written by nurses were collected. Then, monthly numbers of clinical malaria on outpatient books were recalculated. In terms of "clinical malaria", the cases were counted according to the official definition in the HIS determined by the government as "all patients with symptoms of malaria (fever > 37.5°C, a history of fever, and clinical evidence of malaria) who are treated by anti- malarial drugs" [Annual Health Report 2006 Solomon Islands. National Health Statistics Office, Division of Policy and Planning, Ministry of Health, Solomon Islands. 2007 (unpublished)]. The definition, however, was often misunderstood by nurses as confirmed cases and only fever cases, which can potentially cause the misreporting of clinical malaria cases.

As for government data to compare with clinics' data in the outpatient books, the unpublished data of clinical malaria in HIS were graciously provided by the Ministry of Health of the government in SI. Regarding the accuracy of data processing by the government following the submission of the paper copies of handwritten monthly reports from clinics to the government, it was verified that government correctly input the numbers of cases in the copies of monthly reports into their HIS system. It was because that all numbers of monthly cases in government statistics were the same as correspondent numbers of cases in the monthly reports of clinics. This means, in order to evaluate whether the data accurately reflect the true burden of clinical malaria, this study could focus on only the accuracy of reporting by nurses filling in the reports, not the accuracy of the following data processing by the government.

Next, some factors such as clinic staffing and record keeping which were potentially related to the data discrepancy between clinics' outpatient books and government statistics were estimated. According to the monthly records in the outpatient books, the following variables were estimated: (a) average number of patients (per nurse and day), which is as a proxy of nurses' busyness; (b) number of illegible writing (per 100 patients); (c) number of omitted data regarding clinical diagnosis (per 100 patients); (d) slide confirmation (per 100 patients), and (e) treatment (per 100 patients). In (b), the illegible handwriting was counted, in case that the handwriting and the case could not be identified by both the author and nurses of the clinic. Also, (c) omitted data of diagnosis, (d) confirmation, and (e) treatment, were counted respectively, when there was no record of patients in the outpatient books. Furthermore, after pre-testing for two clinics to examine the validity of the interview thorough local interviewers, some variables were examined by the structured interviews to nurses who had worked in 2007 in all eight clinics: (f) percentage of registered nurses among both registered nurses and nurse aides; (g) whether monthly report was filled in by registered nurses; (h) use of tally sheets; (i) daily count of malaria cases; (j) whether correct definition of "clinical malaria" was shared by all nurses, and (k) weekly meeting of nurses and microscopists. For (g) to (k), were asked in a scale of four: almost always, often, sometimes, and almost never. With regard to (h), tally sheets are the sheets with a lot of blank circles divided into ten to help nurses to count, which is distributed by the government to all clinics. Variable (i) was included since some clinics were suspected to count a lot of cases on weekly or monthly basis, which might cause miscounting and miscalculation. Last, (l) clinic level (i.e., "Area Health Clinic (AHC)" for upper two clinics given more resources such as nurses by the government and other six "Urban Health Clinic (UHC)") and (m) rainy season (from November to March), as a proxy of nurses' busyness besides (a), were also included. (a)-(f), (g)-(k), and (l)(m) were respectively continuous, ordinal, and categorical variables.

### Data analysis

For the analysis of the data discrepancy between clinics' outpatient books and government statistics and the association between the discrepancy and related variables ((a)-(l)), monthly data of the discrepancy and variables were used, because the values differed among months even in the same clinics. Therefore, the original sample size was 96, since each eight clinic has twelve values from January to December in 2007.

The magnitude of data discrepancy between clinics' data and corresponding government data were computed as:

| (Monthly number of clinical malaria cases in government statistics/Monthly numbers of clinical malaria cases in clinics' outpatient books) - 1 | (%)

The clinics' data can be used as the reference which is more reliable than government data, because the discrepancy may be caused when nurses filled in the monthly report to the Ministry of Health according to clinics' data in outpatient books. At the same time, the monthly "direction" of data discrepancy (i.e., "positive" (government data is larger than clinics' data) or "negative" (government data is smaller than clinics' data)) were recorded.

Then, the mean of the monthly data discrepancy and variables among eight clinics in 2007 were compared using repeated measures analysis of variance (ANOVA) for normally distributed variables (i.e., (b) illegible handwriting and (e) omitted data of treatment) and Friedman's ANOVA test for non-normally distributed variables (i.e., discrepancy and (a) average numbers of patients (per nurse and day), (c) omitted data of diagnosis, (d) omitted data of slide confirmation, (f) registered nurses (%)) and all ordinal variables (g)-(k). Normality of was tested by Skewness/Kurtosis test. If the overall P value obtained from repeated measures ANOVA and Friedman test was significant, then post hoc multiple comparisons were performed by using Tukey-Krammer pairwise method.

Finally, the associations between the monthly data discrepancy and measured variables were evaluated by a multiple regression analysis, after checking of the assumptions such as normality, overfitting, and multicollinearity. Data was entered, processed and analysed using Stata, version 10.0 (Stata cooperation, College Station, USA) [9]. P < 0.05 was considered statistically significant for all tests.

## Results

### Data Discrepancy between clinics' outpatient books and government statistics in clinical malaria case reporting

The monthly data discrepancy between clinics' data recorded in outpatient books and government statistics in clinical malaria case reporting stratified by eight clinics at Honiara are presented in Table 1. Descriptive numbers of clinical malaria cases in both outpatient books and government statistic are also included in the table. "Clinical malaria" was based on the official definition by the government as "all patients with symptoms of malaria (fever > 37.5°C, a history of fever, and clinical evidence of malaria) who are treated by anti- malarial drugs" [Annual Health Report 2006 Solomon Islands. National Health Statistics Office, Division of Policy and Planning, Ministry of Health, Solomon Islands. 2007 (unpublished)]. The mean of the discrepancy was 21.2% (standard error: 3.0%). The discrepancy significantly differed among eight clinics (Friedman's ANOVA P < 0.001, Friedman's chi-square 30.5). The post hoc comparison indicated that Mbokona clinic had significantly greater discrepancy than other six clinics except Kukum clinic.

**Table 1:** Descriptive monthly numbers of clinical malaria cases and estimated monthly data discrepancy between clinics' outpatient books and government statistics in clinical malaria case reporting, stratified by eight clinics, Honiara, Solomon Islands, 2007.

	Clinics									
			
Monthly numbers of clinical malaria cases (Mean (Standard Error))	Kukum	Mataniko	Mbokona	Mbokona-vera	Naha	Rove	Vura	White river	Annual total cases of clinical malaria in eight clinics	
Clinics' outpatient books (i)	297.3 (30.8)	230.5 (14.5)	46.3 (3.5)	180.3 (15.8)	148.6 (7.1)	307.7 (18.7)	85.3 (10.3)	74.2 (6.5)	15840	
Government statistics (ii)	276.8 (42.1)	223.2 (16.5)	49.6 (5.3)	155.4 (14.9)	156.7 (11.2)	304.5 (26.6)	80.3 (10.5)	73.6 (6.9)	16411	

Monthly discrepancy (Mean (Standard Error))	Kukum	Mataniko	Mbokona	Mbokona-vera	Naha	Rove	Vura	White river	Total	P-value^╙^

Magnitude of data discrepancy | ii/i -1 |* (%)	37.9 (12.6)	9.4 (2.0)	60.4 (14.2)	20.8 (3.5)	16.1 (3.0)	8.6 (3.2)	11.7 (3.3)	5.1 (1.7)	21.2 (3.0)	< 0.001
Direction of the data discrepancy ii/i -1 ** (%)	2.4 (17.0)	-3.3 (6.6)	23.4 (12.4)	-12.5 (5.3)	5.6 (3.8)	-2.4 (3.2)	-6.1 (6.2)	-1.3 (2.3)	-0.7 (3.7)	< 0.001

(n = 12 per clinic, n = 96 in total)

* |(Monthly number of clinical malaria cases in government statistics/Monthly numbers of clinical malaria cases in clinics' outpatient books) - 1|

** (Monthly number of clinical malaria cases in government statistics/Monthly numbers of clinical malaria cases in clinics' outpatient books) - 1

"Positive direction" means that government data is larger than clinics' data, and "negative direction" means that government data is smaller than clinics' data.

╙ Friedman's ANOVA test

When the "positive" (government data is larger than clinics' data) and "negative" (government data is smaller than clinics' data) directions of the data discrepancy were considered, the average of the discrepancy was -0.7% (Standard Error: 3.7%). The discrepancy with positive and negative direction also significantly differed among clinics (Friedman's ANOVA P < 0.001, Friedman's chi-square 41.0). The post hoc comparison, however, indicated that no clinic had significantly greater discrepancy than other seven clinics.

### Variables potentially related to the data discrepancy in clinical malaria case reporting

Table 2 shows the descriptive characteristics of monthly variables potentially related to the data discrepancy in malaria case reporting made in eight clinics. All variable significantly differed among clinics, such as (a) average numbers of patients (Friedman's ANOVA P < 0.001, Friedman's chi-square 144.0), (b) illegible handwriting (Repeated measures ANOVA P < 0.01, Degree of Freedom (error) 74, F 12.8), (c) omitted data of diagnosis (Friedman's ANOVA P < 0.001, Friedman's chi-square 28.4), (d) omitted data of slide confirmation (Friedman's ANOVA P < 0.01, Friedman's chi-square 9.48), (e) omitted data of treatment (Repeated measures ANOVA P < 0.001, Degree of Freedom (error) 74, F 40.2), (f) registered nurses (%) (Friedman's ANOVA P < 0.001, Friedman's chi-square 144.0), (g) registered nurse filled in Reports (Friedman's ANOVA P < 0.001, Friedman's chi-square 59.0), (h) use of tally sheets (Friedman's ANOVA P < 0.001, Friedman's chi-square 54.6), (i) daily counts of cases (Friedman's ANOVA P < 0.001, Friedman's chi-square 81.6), (j) shared definition of clinical malaria (Friedman's ANOVA P < 0.001, Friedman's chi-square 79.1), and (k) weekly meeting by nurses and microscopists (Friedman's ANOVA P < 0.001, Friedman's chi-square 71.4).

**Table 2:** Descriptive characteristics of variables potentially related to the data discrepancy between clinics' outpatient books and government statistics in clinical malaria case reporting made in each month in eight clinics, Honiara, Solomon Islands, 2007.

**Continuous Variables**				Mean (Standard Error)	P-value╙
(a) Average numbers of patients (per nurse and day)				24.1 (0.9)	< 0.001^§^
(b)Illegible handwriting (per 100 patients)				6.2 (0.3)	0.01**
(c)Omitted data of diagnosis(per 100 patients)				3.7 (0.2)	< 0.001^§^
(d)Omitted data of slide confirmation(per 100 patients)				6.2 (0.4)	< 0.01^§^
(e)Omitted data of treatment (per 100 patients)				3.2 (0.2)	< 0.001 **
(f) Registered nurses among all nurses and nurse aides (%)				34.6 (0.9)	< 0.001^§^
**Ordinal Variables**				No. (%)	P-value^╙^
	(Almost) Never	Sometimes	Often	(Almost) Always	
(g)Registered nurse filled in Reports	19(19.8%)	43(44.8%)	0(0%)	34(35.4%)	< 0.001^§^
(h)Use of tally sheets	19(19.8%)	31(32.3%)	0(0%)	46(47.9%)	< 0.001^§^
(i)Daily counts of cases	42 (43.8%)	27(28.1%)	18(18.8%)	9 (9.4%)	< 0.001^§^
(j)Shared official definition of clinical malaria	59 (61.5%)	0(0%)	15 (15.6%)	22 (22.9%)	< 0.001^§^
(k)Weekly meeting by nurses and microscopists	50 (52.1%)	7 (7.3%)	12 (12.5%)	27 (28.1%)	< 0.001^§^

**Categorical variables**				No. (%)	
(l)Clinic level*	Area Health Clinic (AHC)		Area Health Clinic (AHC)		
	72(75%)		24(25%)		-
(m)Rainy season	Seven months (from November to March) 56(58.3%)				-

(n = 96 in total, n = 12 per clinic).

(Alphabet) is correspondent to the text.

╙ P value comparing the mean among eight clinics

** Repeated measure analysis of variance (ANOVA) for normally distributed variables

^§ ^Friedman's ANOVA test

† Tally sheets are the sheets with a lot of blank circles divided into ten to help nurses to count cases by marking circles according to the records in outpatient books. They were distributed by the government to all clinics.

*Two clinics, Kukum and Rove, are upper clinics called Area Health Clinics (AHC) given more human and material resources by the government, compared to other six clinics called Urban Health Clinics (UHC).

The post hoc comparison showed that Mbokona clinic had significantly larger (b) illegible handwriting and (e) omitted data of treatment than all other seven clinics, and (d) omitted data of slide confirmation than six clinics except Rove clinic. Also, Mbokona clinic had significantly smaller value in (h) use of tally sheets than all other clinics. White river clinic had significantly higher value on (j) shared definition of clinical malaria, and (k) weekly meeting by nurses and microscopists, compared to other clinics. Regarding (g) registered nurse filled in reports, White river and Rove clinic had significantly larger values than remaining six clinics. Rove clinic had significantly higher (a) average numbers of patients than other six clinics except Kukum clinic. There was a significant value of (f) registered nurses (%) in Mbokonavera clinic, compared to all other clinics. As for other values, there was no apparent significance among clinics.

### Association between data discrepancy in clinical malaria case reporting and potentially related variables

Next, the results of multiple regression for the relationship between the monthly data discrepancy in the reporting of clinical malaria and potentially variables related to the discrepancy are presented in Table 3. In order to minimize non-normality and avoid heteroscedascity, the data discrepancy of clinical malaria, estimated by a ratio of | (Monthly number of clinical malaria cases in government statistics/Monthly numbers of clinical malaria in clinics' outpatient books) - 1 | (%), were transformed to natural logarithms. Regarding the model selection, initially full model with all variables was prepared, due to few past studies providing prior information for the appropriate model. And then, three variables, such as (g) whether registered nurse filled in reports, (i) daily counts of cases, and (k) whether weekly meetings occurred, violating the multicollinearity and overfitting for the regression were removed. Multicollinearity was suspected when Variance Inflation Factor (VIF) was over ten, and overfitting was considered when adjusted R square turned to decrease. As the result, there were significant associations between the data discrepancy and four variables: (a) average number of patients (coefficient: 0.05, 95%CI: 0.31, 0.07), (b) illegible handwriting (coefficient: 0.09, 95%CI: 0.04, 0.15), (h) use of tally sheets (coefficient:-0.38, 95%CI: -0.54, -0.22), and (m) clinic level (coefficient:-0.48, 95%CI:-0.89,-0.06). The model selection was complementarily performed by using stepwise regression, which indicated that the four variables always had significant relationships to the data discrepancy under any expectable models including the upper one. With those exceptions, apparent associations between discrepancy and other variables were not observed.

**Table 3:** The association between data discrepancy (between clinics' outpatient books and government statistics in clinical malaria case reporting) and variables, using multiple regression.

**Variable**	**Coefficient**	**(95%CI^§^)**	**P-value**
**(a) Average numbers of patients (per nurse and day)**	**0.05**	**(0.31,0.07)**	**< 0.001**
**(b) Illegible handwriting (per 100 patients)**	**0.09**	**(0.04,0.15)**	**0.017**
(c) Omitted data of diagnosis(per 100 patients)	0.04	(-0.06,0.13)	0.642
(d) Omitted data of slide confirmation(per 100 patients)	0.02	(-0.03,0.07)	0.567
(e) Omitted data of treatment (per 100 patients)	0.06	(-0.05, 0.18)	0.447
(f) Registered nurses among all nurses and nurse aides (%)	-0.01	(-0.02, 0.01)	0.398
**(h) Use of tally sheets**^†^	**-0.38**	**(-0.54, -0.22)**	**< 0.001**
(j) Shared official definition of clinical malaria by nurses	-0.07	(-0.28, 0.13)	0.379
**(l) Clinic level**╙	**-0.48**	**(-0.89, -0.06)**	**0.019**
(m) Rainy season∏	-0.05.	(-0.34, 0.24)	0.75

(n = 92)

(Alphabet) is correspondent to the text.

Significant variables are in bold. ^§^Confidence Interval (Adjusted R squared: 0.67)

(a)-(f) are continuous, (h)(j) are ordinal, and (l)(m) are categorical variables. The following variables were removed because of the violation to multicollinearity and overfitting: (g) whether registered nurse filled in reports, (i) daily counts of cases, and (k) whether weekly meetings occurred.

**†**Tally sheets are the sheets with a lot of blank circles divided into ten to help nurses to count cases by marking circles according to the records in outpatient books. They were distributed by the government to all clinics.

╙Two clinics, Kukum and Rove, are upper clinics called Area Health Clinics (AHC) given more human and material resources by the government, compared to other six clinics called Urban Health Clinics (UHC).

∏ From November to March

## Discussion

### Magnitude of data discrepancy between clinics' outpatient books and government statistics in clinical malaria case reporting

This study demonstrated insufficient accuracy of clinical malaria case reporting through significant gaps between clinics' records and government statistics in HIS system. The average data discrepancy was large at 21.2%, indicating that one fifth of the numbers was over or underestimated when nurses reported the cases to the Ministry of Health of the government in SI. This finding suggests that there could be numerous reporting errors made by nurses in local clinics.

Moreover, when the "positive directions" (government data is larger than clinics' data) and "negative directions" (government data is smaller than clinics' data) of the data discrepancy were estimated, the average discrepancy were -0.7%, which means there were almost the same chances of positive and negative directions to be cancelled out. As a result, the seeming discrepancy summarizing the monthly numerical superiority of the cases between clinics and the government was much smaller than the true discrepancy, namely 21.2%. This suggests that certainly nurses made a lot of reporting errors leading to the actual huge discrepancy; however, occasionally such errors would have randomly occurred among nurses, thereby reducing the magnitude of the data discrepancy. One of the possible explanations to both directions is nurses' misunderstanding of the definition of clinical malaria. According to the interview data, 62.2% of all nurses (n = 45) misunderstood the definition. Among the 62.2% of all nurses, 42.9% of the nurses overestimated clinical malaria cases, because they included fever cases into clinical malaria, which caused the positive direction of the discrepancy. By contrast, 57.1% of the nurses confused clinical malaria with slide-confirmed malaria, which caused the negative direction of the discrepancy. An example is that, in Mbokonavera clinic having -12.5% of the discrepancy (table 1), all six nurses except one registered nurse confused clinical malaria with confirmed malaria. This suggests that government should give training to nurses about the official definition of clinical malaria for the accurate reporting, since no action for the misunderstanding of nurses had been taken in SI.

Thus, these results propose that clinical malaria case reporting at the local clinic levels urgently needs improvement for the accurate surveillance of malaria. In practice, among eight clinics in Honiara, Mbokona clinic ought to be particularly paid attention to, due to the significantly greater discrepancy than other clinics except Kukum clinic.

### Factors related to the data discrepancy

This study found that the use of tally sheets, lower numbers of patients, readable handwriting, and clinics at the upper level (Area Health Clinic (AHC)) were associated with the decrease in the data discrepancy between clinics' outpatient books and government statistics in clinical malaria case reporting.

First of all, tally sheets can be one of the most reasonable and practical solution to accurate reporting. The Ministry of Health distributed tally sheets with a lot of blank circles divided into ten, which helped nurses to correctly count the cases by marking circles according to the records in outpatient books. Some nurses, however, lost the tally sheets, which may easily cause miscalculations. Particularly, Mbokona clinic with significantly smaller value in the variable of use of tally sheet than all other clinics never used the sheets in 2007. Thus, in Honiara, the usage of tally sheets should be encouraged in especially Mbokona clinic, aiming to reduce the significant data discrepancy of the clinic.

Next, the increase in numbers of patients per nurse and day negatively associated with the accuracy of the reporting. The number of patients would be a proxy of nurses' busyness, since outpatients care may be their main work, even though they have additional work, such as home-visiting, meeting, and administrative things. When nurses do not have enough time to fill in the books due to many patients, they are likely to make more mistakes. Increasing the numbers of nurses to reduce the work-load per person could be a solution in the future. With regard to clinics in Honiara, Rove clinic, one of Area Health clinic (AHC) given more resources by the government, should be focused on, because the clinic had significantly greater number of patients than other clinics except Kukum clinic, the other AHC. At the time, however, for the accurate reporting, an increase in the percentage of registered nurses might be unnecessary, since the result indicated that the percentage of registered nurses was insignificantly related to the discrepancy. One possible reason is that the good data management to reduce the discrepancy depends on the skill of some chief registered nurses directing other nurses rather than the number of registered nurses. This implies that even registered nurses have different levels of clinical management skill, and then some clinics could have the better outcomes in months when careful registered nurses were moved in.

In addition, the illegible handwriting in outpatient books was a significant problem that led to an increase in the discrepancy. In contrast, omission of data about diagnosis, investigation, and treatment were also often seen in the books, but they were not significant factors related to the frequency of the discrepancy. Perhaps this is because even if nurses omit specific data (e.g., diagnosis), they can successfully distinguish clinical malaria cases according to other data (e.g., treatment) of the same patients. Moreover, unexpectedly the illegible handwriting and omission of data did not have strong correlations to nurses' work-load, such as the numbers of patients. This suggests other potential factors related to the illegible handwriting and omission. For a practical immediate solution, easier recording, like a chart with a scale allowing nurses to fill in just numbers into spaces of diagnosis, investigation, and treatment on the outpatient books, may prevent the illegible handwriting and omission. Among eight clinics in Honiara, Mbokona clinic had significantly larger illegible handwriting, which means that Mbokona clinic would need special efforts to improve their handwriting in order to reduce the huge data discrepancy.

Last, it was observed that two upper level clinics (AHC), Kukum and Rove, given more resources by the government are likely to decrease the data discrepancy. This point, however, had debatable results: as mentioned above, Rove clinic had significantly larger patients related to the increase in the discrepancy. One of the reasons of this contradiction could be unknown potential confounders which associate with clinic level.

Therefore, this study strongly implied that the central government and Honiara city council in SI should strengthen the supervision and training to nurses regarding clinical malaria reporting, in order to achieve the reliable surveillance and malaria elimination. In the supervision and training, encouragement of the use of tally sheets, legible handwriting, and the understanding of the official definition of clinical malaria ought to be emphasized. Also, further efforts to avoid large numbers of patients per a nurse and day in the current health system would be important. Such supervision and training to nurses should be introduced in some stages of their careers.

### Limitations

This study potentially has memory bias, because some variables, such as use of tally sheets, and proportion of registered nurses were based on the memory of the nurses who have worked since 2007 in the same clinics. Also, the possibility that some clinics have additional outpatient books besides the collected ones cannot be denied. Furthermore, there can be some factors, like nurses' clinical diagnostic skill, which might correlate with the data discrepancy. In the interviews of this study, actually 38.9% of nurses recognized the insufficiency of their own diagnostic skills. This study, however, focused on the reporting accuracy, which is a separate issue from diagnostic accuracy as mentioned in background. In addition, this result cannot be generalized to other provinces in SI, because the study area is only in Honiara, the capital of SI, which has more human and material resources than other provinces.

Finally, even though clinical malaria case reporting HIS could be improved, HIS is a kind of passive case detection (PCD), which could underestimate the burden of malaria because of the patients' poor access to clinics and self-medication. For the estimation of true burden, further study such as active case detection (ACD) will be needed [10-12].

## Conclusion

This study found insufficient accuracy of clinical malaria case reporting through significant gaps between clinics' records and government statistics. The average monthly data discrepancy was large with 21.2%, which shows one fifth of the numbers were over- or underestimated when nurses reported the cases to the Ministry of Health. This suggests that clinics made numerous errors leading to such huge discrepancy.

Moreover, the study also suggests that the high numbers of patients, disuse of tally sheets, illegible writing, and some clinics given fewer potential resources by the government are significantly related to the frequency and magnitude of the data discrepancy. Additionally, in Honiara, certain clinics should be focused on to improve the reporting accuracy because of the significantly problematic status among clinics in some respects.

In the end, the clinical malaria case reporting at local clinics urgently needs improvement for malaria surveillance and the disease elimination in SI.

## Competing interests

The author declares that they have no competing interests.

## Authors' contributions

The author initiated the study, led the field survey, analysed data and drafted the manuscript.
